# Effect of the Go4it multidisciplinary group treatment for obese adolescents on health related quality of life: a randomised controlled trial

**DOI:** 10.1186/1471-2458-13-939

**Published:** 2013-10-08

**Authors:** Geesje H Hofsteenge, Peter JM Weijs, Henriette A Delemarre-van de Waal, Maartje de Wit, Mai JM Chinapaw

**Affiliations:** 1 Department of Nutrition & Dietetics, Internal Medicine, VU University Medical Center Amsterdam, De Boelelaan 1117, Amsterdam, HV 1081, The Netherlands; 2 EMGO Institute for Health and Care Research, VU University Medical Center Amsterdam, Amsterdam, The Netherlands; 3 Department of Nutrition & Dietetics, Amsterdam University of Applied Sciences, Amsterdam, The Netherlands; 4 Department of Paediatrics, Leiden University Medical Center, Leiden, The Netherlands; 5 Department of Paediatrics, VU University Medical Center Amsterdam, Amsterdam, The Netherlands; 6 Department of Medical Psychology, EMGO Institute for Health and Care Research, VU University Medical Center Amsterdam, Amsterdam, The Netherlands; 7 Department of Public and Occupational Health, VU University, Amsterdam, Amsterdam, The Netherlands

**Keywords:** Adolescents, Obesity, Health related quality of life, Randomized controlled trial

## Abstract

**Background:**

We developed a Dutch outpatient multidisciplinary group treatment (Go4it) for obese adolescents, including cognitive behavioural therapy and education on healthy dietary and physical activity behaviour. This study examined the effect of Go4it on Health Related Quality of Life (HRQoL).

**Methods:**

At our outpatient paediatric obesity clinic, obese adolescents (n = 122, 11–18 years) were randomly assigned to 1) Go4it, 7 sessions with an interval of 2 weeks or 2) current regular care consisting of referral to a dietician in the home care setting (controls). Linear mixed model analysis was performed to evaluate the intervention effects on HRQoL at 6 and 18-month follow-ups. HRQoL indicators included the Child Health Questionnaire, the Paediatric Quality of Life Inventory™ Version 4.0 (PedsQL™4.0), and the Body Esteem Scale (BES).

**Results:**

In total, 95 adolescents (Go4it 57, controls 38) were included in the current analysis with a mean age of 14.5 ± 1.7 and mean BMI-SDS of 2.9 ± 0.5. At baseline, all participants experienced lower levels of physical and psychosocial well-being compared to a normal weight reference group. At the 18 month follow-up, we found small but beneficial intervention effects on all subscales of the PedsQL™4.0 and BES questionnaires. Two subscales improved significantly; i.e., physical health (*between group difference* 5.4; 95%CI: 0.3; 10.6), and school functioning (*between group difference* 7.4; 95%CI: 1.6; 13.2).

**Conclusion:**

Obese adolescents experienced lower HRQoL than their healthy peers. The Go4it intervention had small beneficial effects on HRQoL compared to the current regular care practices for obese adolescents.

**Trial registration:**

Netherlands Trial Register: ISRCTN27626398, METC number: 05.134 (WMO, monocenter).

## Background

Obesity is one of the most common chronic disorders in children and adolescents and its prevalence continues to increase rapidly. Of Dutch children aged 2–21 years old, 13-15% were overweight and 2% were classified as obese in 2009 [1]. Obesity is also one of the most stigmatizing and least socially accepted conditions in childhood [2]. The most widespread consequences of adolescent obesity are psychosocial [3]. Early adolescence may be a particularly vulnerable period for reductions in Health Related Quality of Life (HRQoL) in overweight/obese youth, since heightened awareness of social exclusion and participation limitations occur [4,5]. As in a not overweight population there are adolescents with a higher or lower quality of life (QoL).

The World Health Organization defines QoL as “individuals’ perceptions of their position in life in the context of the culture and value systems in which they live and in relation to their goals, expectations, standards and concerns [6]”. It incorporates the person’s physical health, psychological state, level of independence, social relationships, and their relationship to salient features of their environment in a complex way [6].

Negative self-image and a reduced self-esteem in overweight children, which can begin as early as the age of five, can ultimately result in sadness, loneliness, nervousness and high-risk behaviour’s as children develop into obese adolescents [7]. Obesity is also predictive of being the victim of bullying in adolescents [8]. These widespread psychosocial consequences of childhood obesity in adolescents impair their HRQoL [2]. Schwimmer et al. showed that obese children and adolescents (5–18 years old), who where newly referred to the clinic, reported significantly lower QoL in all domains compared with normal weight children and adolescents [2].

Notwithstanding the high prevalence of child obesity, little evidence exists regarding effective child obesity treatments [9]. Most studies included children age 7–12 years old, and only a few studies have evaluated treatment of adolescent obesity [9-12]. Often, outpatient treatment for the obese is focused on nutrition education and physical activity [9-13]. Wille et al. [14] and Vignolo et al. [15] showed improvement on the effect of their inpatient and outpatient treatment programs not only on diet and physical activity but also on the HRQoL of children aged 6–16 and 6–12 years, respectively. Breat et al. showed promising results of cognitive behavioural modification techniques regarding lifestyle changes in obese children [16]. Based on these positive experiences, and the fact that there is no effective treatment available for this age group, we developed a multidisciplinary group treatment for obese Dutch adolescents (Go4it) [17]. Understanding HRQoL can contribute to a better awareness of the patients’ needs, as well as improve care and treatment.

This study describes the long-term effects of the Go4it group treatment for obese adolescents on HRQoL aspects in a randomised controlled trial. We hypothesised that at baseline our study sample would have lower HRQoL compared to a normal weight reference group. We also hypothesised that the Go4it treatment would have a beneficial effect on HRQoL.

## Methods

### Subjects and design

The present study is a randomised controlled trial evaluating the effect of the Go4it multidisciplinary group treatment for obese adolescents, on HRQoL at six and 18 month follow-ups. Adolescents were referred to the outpatient paediatric obesity clinic of the VU University Medical Center Amsterdam by their general practitioner or school physician. During the first visit one of three paediatric-endocrinologists interviewed all adolescents concerning their medical history, social problems, teasing, weight development, socio-economic status (SES) and ethnicity according to a standard protocol. Subjects were categorised as having a western ethnicity when both parents were Dutch or with at least one parent was born outside the Netherlands, but inside Europe (including former Yugoslavia and Soviet Union), North America, Oceania, Indonesia or Japan. Subjects with at least one parent born in Turkey, Africa, Latin America or Asia were classified as non-western [18]. The physical examination included height, weight, waist circumference, blood pressure and pubertal Tanner stage [19].

The subjects and their parents received an informational brochure about the study. Within two weeks, the research assistant checked their willingness to participate. Subjects were eligible when they met the following inclusion criteria: 1) age 11–18 years; and 2) overweight or obese according to the definition of Cole et al. with gender and age specific cut off values [20]. Exclusion criteria were: not Dutch-speaking, obesity as a result of a known syndrome or organic cause (hypothyroidism), mental retardation, physical limitations (e.g. crutches or wheelchair) and diagnosis of type 2 diabetes mellitus. The research assistant used block randomisation to assign subjects randomly to the intervention (60%) or control group (40%), using SPSS for random selection. This distribution was chosen to recruit a sufficient number of adolescents to start the intervention sessions. Randomisation was stratified for sex and age group (11–14 years old and 15–18 years old). Since adolescents knew to which group they were assigned, participants could not be blinded. The medical ethical committee of VU University Medical Center Amsterdam approved the protocol. Adolescents as well as their parents gave written informed consent.

### Intervention

Go4it is a multidisciplinary group treatment for obese adolescents based on the programs of Braet et al. [16], Epstein et al. [21], and the educational materials of the Dutch Obesity Intervention in Teenagers (DOiT) [22]. During 7 sessions with two-week intervals, the adolescents received education on healthy dietary behaviour, screen behaviour and physical activity. The group size was 8 to 12 adolescents. The first session was focussed on increasing awareness of the current lifestyle. Besides dietary and activity journals, step counters (pedometers) were used to increase awareness of the actual behaviour. Next, adolescents were instructed to set goals with respect to improving their physical activity and dietary behaviour. Additionally cognitive- behavioural therapy characteristics (mainly based on problem-solving techniques) were used, for example, learning how to improve their lifestyle, learning to cope with teasing, to improve self-esteem, and how to maintain energy balance. Go4it works with homework tasks and the education is interactive. Go4it was carried out in an outpatient clinic involving a dietician, psychologist, and a paediatric-endocrinologist. They were also all involved in the development of Go4it.

In addition, two separate parallel sessions for parents were organised corresponding to the first and fourth session of the adolescents. These parental sessions consisted of education concerning healthy dietary behaviour and physical activity, the health risks of overweight and how to support their obese children in improving their behaviour. Four booster group sessions for the adolescents were scheduled 6, 14, 26, and 36 weeks after the 3-months intervention period, in order to encourage the adolescents to maintain or further improve their energy balance behaviour, discuss problems and answer questions. Throughout the program the adolescents remained in the same peer group. Special materials were developed for this program: an information book, a workbook, and a dietary and activity diary. In addition, specific worksheets for every session were developed. The control group received the current regular care in the Netherlands, consisting of referral to a dietician in the home care setting. Adolescents had to make an appointment themselves. Reasons for non-compliance were collected by phone and questionnaire. Details of the intervention have been published elsewhere [17].

### Study protocol

After an overnight fast, subjects attended the outpatient clinic. Height was measured with an accuracy of 0.1 cm with an electronic stadiometer (KERN 250D, De Grood Metaaltechniek, Nijmegen, The Netherlands). Body weight was measured (in underwear) within 0.1 kg with a calibrated electronic flat scale (SECA 861, Schinkel, Nieuwegein, The Netherlands). Weight and height were used to calculate BMI (kg/m^2^). For calculation of BMI standard deviation scores (BMI-SDS) or z-scores, a reference database of Dutch children was used (http://www.growthanalyser.org; version 3.5). The researcher conducted all measurements. The adolescents filled in the questionnaires (PedsQL™4.0, CHQ and the BES) in the morning during a visit to the obesity clinic. The adolescents completed the questionnaires’ independently and the research assistant entered the data. Baseline measurements took place between November 2006 and August 2008. Measurements were repeated after 6 and 18 months.

### Questionnaires

HRQoL was examined using the generic reliable and validated Paediatric Quality of Life Inventory™ Version 4.0 (PedsQL™4.0) [23,24] and the generic Child Health Questionnaire (CHQ) [25]. PedsQL™4.0 assesses physical, emotional, social and school functioning, while CHQ assesses physical, behavioural, mental and social functioning.

The 23-item PedsQL™4.0 questionnaire encompasses physical functioning (eight items), emotional functioning (five items), social functioning (five items) and school functioning (five items). A 5-point Likert scale was used for response (0 = never a problem; 4 = almost always a problem). Items are reversed scored and linearly transformed to a 0–100 scale, so that higher scores indicate better HRQoL. A total scale score and physical and psychosocial health summary scores were also calculated [23,25].

The psychometric properties of the Dutch translation of the PedsQL™4.0 questionnaires has been well established by van Engelen et al. [26]. Their study population consisted of 496 healthy schoolchildren aged 5–7 years (n = 92), 8–12 years (n = 219) and 13–18 years (n = 185). There are no known data for weight or BMI [26]. Reliability of the PedsQL™4.0 was good in our sample (Total score: Cronbach’s α = 0.87; Physical health score: Cronbach’s α = 0.79; Psychosocial health score: Cronbach’s α = 0.81).

The CHQ Child Form 87 questionnaire encompasses 12 domains of which each item contains 4, 5 or 6 response alternatives. Per scale, the items are summed up (some recoded/recalibrated) and transformed to a 0 (worst possible score) to 100 (best possible score) scale [25]. In this study, a physical summary scale was computed, as the mean of the items in the CHQ-subscales of physical functioning, role/social limitations-physical, general health perceptions and bodily pain. Further, we computed a psychosocial summary scale, as the mean of the items in the CHQ-subscales of role/social limitations-emotional, role/social limitations-behavioural, self esteem, mental health and general behaviour. The psychometric properties of the Dutch translation of the CHQ questionnaire has been well established in a population of 466 schoolchildren (age 9–17 years) by Raat et al. [27]. In these children, allergies (17%), eczema (8%), migraines (6%) and asthma (5%) were the most prevalent reported conditions. Again, there is no known information for weight or BMI [27]. Reliability of both the physical and psychosocial summary scales was good in our sample (Cronbach’s α = 0.81 and 0.93).

### Body esteem scale

Body esteem is an essential part of psychosocial wellbeing in overweight adolescents, but not covered in PedsQL ™4.0 nor CHQ. The Body Esteem Scale (BES) is a validated questionnaire [28] on general feelings about appearance, weight satisfaction and evaluations of attributions to others about one’s body and appearance. Mean scores range from 0 (worst possible score) to 4 (best possible score), with higher scores representing a better body esteem.

### Sample size

The sample size calculation is based on the primary outcome of the trial i.e. BMI-SDS: the number of participants needed to detect a difference in BMI-SDS of 0.29 (10%) between the intervention and control group after 18 months with a standard deviation of 0.5 is 43 subjects per group with an alpha of .05 and a power of .80. A sample of 108 persons (n = 54 per group) was required taking into account a dropout rate of 25%.

### Statistical analysis

Baseline characteristics were analysed by t-tests for continuous variables and Chi-square tests for categorical variables. Scoring and substitution of missing values was performed according to existing manuals. In the case of 50% or less missing per subscale, substitution by the mean was used [24,25,28]. Subscales with higher amounts of missing values were considered missing resulting in a varying amount of participants included in the analyses. Group comparisons were performed according the intention-to-treat principle whereby all subjects were analysed in the group to which they were initially assigned. Linear mixed models were applied to assess the effect of the intervention over time. A random intercept and a random slope with time were assumed. Age-, sex- and, ethnicity adjusted analyses were performed with intervention as the categorical variable and time as the continuous variable; an interaction term for intervention and time was also included. B coefficients (*between group difference*), 95% confidence intervals and *p* values were calculated. This approach has increased statistical power as it accounts for within-person correlation over time and allows different numbers of assessments. All assessments, including baseline, were taken into account. A *p* value of < 0.05 was considered statistically significant. Effect modification by sex, age and ethnicity was checked by adding an interaction term between group allocation and the potential moderator. For effect modification, a *p* value of < 0.1 was considered statistically significant. Finally, effect size estimations (Cohen’s *d*) were calculated in order to decide whether statistical differences were clinically relevant. Effect sizes relate to the difference in mean scores to the dispersion of the scores: [Mean baseline – Mean follow-up]/pooled standard deviation [29]. Following Cohen’s *d* effect size, *d* = 0.2 was taken to indicate a small effect size, *d* = 0.5 a moderate effect size, and *d* = 0.8 a large effect size [30]. All analyses were performed using SPSS software (version 18 · 0, 2009 SPSS Inc., Chicago, Illinois, USA).

## Results

### Subjects

**Figure 1:**
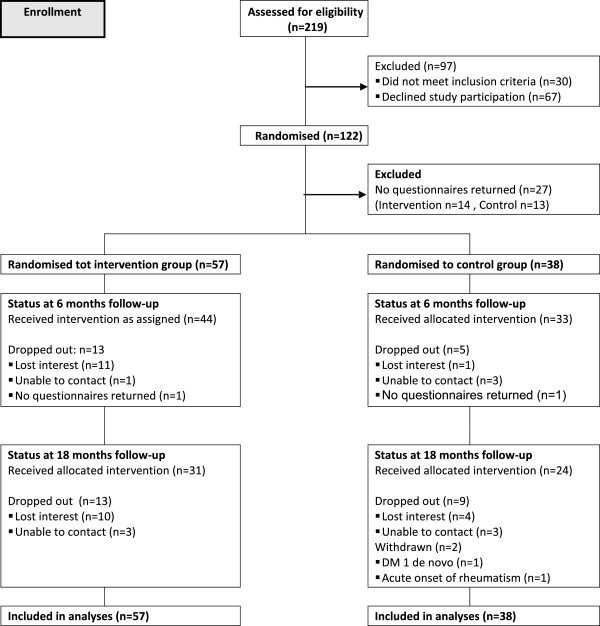
Flowchart of the intervention and control subjects in the randomized controlled trial.

Figure 1 shows the consort diagram for the Go4it trial. Of the 219 adolescents who were assessed for eligibility, 122 consented to the trial and were randomly (60:40) assigned to the intervention (n = 71) and control group (n = 51). At 18 months two subjects from the control group were excluded from the analyses, one developed type 1 diabetes and another was diagnosed with acute rheumatism. Linear mixed models were applied whereby all subjects were analyzed in the group to which they were randomly assigned.

Of the 122 randomised adolescents, 95 completed all questionnaires at baseline. Table 1 presents anthropometric and demographic characteristics of these 95 study participants. No significant differences were found in baseline characteristics between Go4it and control group, nor between the 95 study participants and the 27 adolescents who did not fill in the baseline questionnaires.

**Table 1:** Baseline demographic and anthropometric characteristics of Go4it and control group participants

	**Intervention group**	**Control group**	***p***
	**(n = 57)**	**(n = 38)**	
Age, y ^1^	14.6 *(1.6)*	14.5 *(1.7)*	*0.8* ^*a*^
Sex (n)			
Girls/Boys	29/28	24/14	*0.2* ^*a*^
Height, cm	168.4 *(10.1)*	165.3 *(8.3)*	*0.1* ^*a*^
Weight, kg	94.6 *(18.6)*	91.9 *(17.9)*	*0.5* ^*a*^
BMI, kg/m2	33.1 *(4.4)*	33.4 *(5.0)*	*0.8* ^*a*^
BMI-SDS	2.9 *(0.4)*	2.9 *(0.5)*	*0.8* ^*a*^
Ethnicity (n)			
Western/Non-western	29/28	14/24	*0.2* ^*b*^
Teasing (n)			
Yes/No	23/32	17/18	*0.5* ^*b*^
Missing^2^	3	3	
Education adolescents (n)			
Low ^3^	44	28	*0.7* ^*b*^
High ^4^	12	9	
Missing^2^	1	1	

^*1*^* Data are (means (sd)), unless otherwise indicated*.

^2^ No information available on item in question, ^a^*t*-test, ^b^ Chi Square.

^3^ primary school, or lower secondary education,

^4^ upper secondary education.

### Physical and psychosocial well being

Physical and psychosocial well-being scores and between group differences at 6 and 18 month follow-ups are presented in Table 2. At baseline, participants experienced lower levels of physical and psychosocial well-being scores compared with a healthy reference group from Engelen et al. (PedsQL™4.0) [26] and Raat et al. (CHQ) [27].

**Table 2:** CHQ- CF87 scale -, PedsQL™4.0 and BES mean scores and between group difference between the intervention group and control group at baseline, 6 month and 18 month follow-ups

	**Intervention group**^**2**^			**Control group**^**2**^				
	**Baseline**	**6 month**	**18 month**	**Baseline**	**6 month**	**18 months**	**6 months**	**18 months**
	**Mean ± sd**	**Mean ± sd**	**Mean ± sd**	**Mean ± sd**	**Mean ± sd**	**Mean ± sd**	**B (95% CI)**^**3**^	**B (95% CI)**
**CHQ-CF87**^**1**^								
Physical summary scale	78.2 ± 12.1	80.2 ± 11.2	82.2 ± 10.4	78.3 ± 11.1	82.5 ± 9.9	80.4 ± 11.6	-2.0 (-6.6; 2.5)	2.3 (-2.9; 7.4)
Psychosocial summary scale	80.9 ± 10.1	82.6 ± 10.9	85.1 ± 9.9	81.5 ± 10.0	83.9 ± 8.3	83.5 ± 10.2	-1.5 (-5.9; 2.8)	1.0 (-4.0; 6.0)
**PedsQL**^**tm**^**4.0**^**1**^								
Total score	75.1 ± 12.2	78.5 ± 11.2	81.7 ± 12.0	75.7 ± 10.7	77.9 ± 10.0	77.2 ± 10.5	-0.1 (-3.5; 3.3)	3.8 (-0.2; 7.7)
Physical health	76.5 ± 14.8	83.1 ± 11.8	86.7 ± 11.8	76.4 ± 13.2	78.6 ± 11.9	79.8 ± 11.4	3.6 (-1.0; 8.2)	5.4 (0.3; 10.6)^4^
Psychosocial health	74.7 ± 12.6	76.9 ± 12.0	80.0 ± 12.8	75.4 ± 11.2	77.8 ± 10.2	76.4 ± 11.6	-1.0 (-4.6; 2.6)	3.4 (-0.8; 7.6)
Emotional functioning	74.7 ± 17.2	77.9 ± 15.3	76.1 ± 17.9	74.5 ± 15.8	76.1 ± 12.0	76.1 ± 16.4	1.5 (-4.1; 7.1)	1.8 (-4.8; 8.4)
Social functioning	78.2 ± 15.5	81.8 ± 14.0	87.3 ± 12.4	78.8 ±16.1	82.8 ± 14.0	81.3 ± 13.9	-0.5 (-5.1; 4.1)	2.4 (-3.0; 7.8)
School functioning	71.1 ± 15.3	71.1 ± 15.0	76.6 ± 16.2	73.0 ± 15.4	75.5 ± 15.3	71.7 ± 14.4	-3.2 (-8.1;1.7)	7.4 (1.6; 13.2)^5^
**BES**^**1**^								
Body appearance	1.8 ± 0.7	2.1 ± 0.6	2.2 ± 0.7	1.9 ± 0.8	2.1 ± 0.7	2.2 ± 0.7	0.0 (-0.2; 0.3)	0.1 (-0.1; 0.4)
Weight satisfaction*	1.6 ± 0.7	1.7 ± 0.7	1.7 ± 0.7	1.7 ± 0.6	1.8 ± 0.7	1.8 ± 0.7	0.0 (-0.2; 02)	0.2 (0.0; 0.5)
Body attribution	1.8 ± 0.8	2.1 ± 0.7	2.3 ± 0.8	1.9 ± 1.0	1.9 ± 0.7	2.0 ± 0.6	(0.0; 0.5)	0.3 (0.0; 0.6)

^1^CHQ scores and PedsQL™4.0 range from 0 (worst possible score) to 100 (best possible score) with higher scores representing a better quality of life. BES scores range from 0 (worst possible score) to 4 (best possible score), with higher scores representing a better body esteem.

^2^The numbers of subjects varied at 6 months from 44–45 in the intervention group and from 33–34 in the control group. After the 18 month follow-up the numbers of subjects varied in the intervention group between 34–35 and in the control group from 23–28.

^3^As indicated by unstandardised regression coefficients (B) and 95% confidence intervals (95% CI), adjusted for baseline value, age, sex and ethnicity.

^4^= effect size *d*: 0.7,

^5^= effect size *d*: 0.3.

At the 18 month follow-up we found small but beneficial intervention effects on all subscales of the PedsQL™4.0 and BES questionnaires. Two subscales of the PedsQL™4.0 improved significantly, i.e. physical health (*between group difference* 5.4; 95% CI: 0.3; 10.6) with a clinical effect size of 0.7, and school functioning (*between group difference* 7.4; 95% CI: 1.6; 13.2), with a clinical effect size of 0.3. We found effect modification by sex and ethnicity on a few HRQoL outcomes. The results were inconsistent and for this are reason not presented.

Concerning compliance, 42 (59%) of the 71 subjects in the intervention group, attended at least five Go4it sessions. The reasons for not attending the Go4it sessions included lack of motivation to change dietary habits, lack of belief of parents in their child’s potential success to lose weight, previous unsuccessful dieting experiences, travel distance and the limited time of working parents and schoolchildren. Seven of the 29 subjects who attended less than five sessions never started the Go4it program. At 6 months, 21 (48%) subjects in the control group had never visited a dietician, 4 subjects visited a dietician once, 6 twice, 7 three or more times, and for 6 subjects it is unknown. The main reported reason for not making an appointment was lack of motivation because of previous unsuccessful dieting experiences with or without a dietician [31].

## Discussion and conclusions

This study describes the effectiveness of an obesity treatment program on health related quality of life (HRQoL) aspects in obese adolescents. We found small but beneficial intervention changes on all subscales of the PedsQL™4.0 and BES questionnaires. Two subscales of the PedsQL™4.0, physical health and school functioning, improved significantly in favour of the intervention group. Thus, our low intensive outpatient multidisciplinary group treatment had small but beneficial effects on quality of life of obese adolescents.

### Implications

As we mentioned earlier few studies examined HRQoL among obese adolescents enrolled in outpatient programs and the effects of these programs on HRQoL [14-16]. Most programs targeting obese adolescents mainly focus on weight change [9,13].

In our study we found a beneficial decrease in BMI-SDS at the 6 month follow-up *(between group difference*: -0.10 (-0.23; 0.04) and a significant decrease at the 18 month follow-up (*between group difference*: -0.16; 95%CI: -0.30;-0.02) [31]. We found a significant but low correlation (R = -.359) between change in BMI-SDS and change in physical health (PedsQL™4.0) at the 6 month follow-up, but not at 18 month follow-up.

We also found a significant effect on experienced physical health and school functioning at the 18 month follow up. A possible reason for the improved school functioning may be the cognitive behavioural therapy elements of Go4it focussing on dealing with difficult situations and how to react to teasing. This may have provided them with tools for less distraction and better concentration at school. The improved physical health may also have resulted in fewer days of sick leave and thereby had a positive influence on school attendance. As the CHQ does not tap into school issues, we found no differences there.

### Other studies

Our study is in line with previous studies showing that all mean physical and psychosocial summary scores at baseline were lower in obese adolescents in comparison with healthy peers. Therefore, they experience a lower quality of life [23,26], but similar to those reported in other obese samples [23,32]. In a cross-sectional study, de Beer et al. compared HRQoL in obese Dutch adolescents to age and sex matched normal weight controls (n = 62) [32]. CHQ and PedsQL™4.0 scores of obese adolescents in their sample were less than their normal weight controls and resembled the mean scores in our sample. Also similar scores on both questionnaires’ were found among obese children aged 8–18 years old and among Dutch children suffering form a chronic health condition to those in our sample [26,33].

For the BES, Dutch reference data on obese adolescents are currently not available. In a study by Mendelson *et al*. [28], in which body esteem among healthy Canadian adolescents was measured, adolescents reported mean scores ranging from 1.8 to 3.0. As expected, obese adolescents in our study reported slightly lower mean body esteem scores.

Unlike the two subscales of the PedsQL™4.0, we found no significant intervention effect on the CHQ. The PedsQL™4.0 addresses more serious problems and includes more subscales than the CHQ. The PedsQL™4.0 may therefore be more sensitive to subtle changes.

### Strength and limitations

Most outpatient programs in adolescents that combine education on healthy nutrition and physical activity with cognitive behavioural therapy primarily evaluated effects on weight status. Few have evaluated effects on HRQoL. Other strengths of our study are the randomised controlled trial design, the relatively easy to implement outpatient intervention and the relatively long-term follow up. Another strength is the information from three questionnaires, providing the opportunity to examine intervention effects on various aspects of HRQoL.

A limitation of our study may be selection bias, because participants were obese adolescents referred to a medical obesity outpatient clinic in Amsterdam. Our findings may not be generalisable to the larger group of obese adolescents seen or treated by general health practitioners in smaller cities in the Netherlands. The majority (59%) of our study sample was living in Amsterdam, where almost 50% of the population is of non-western descent, this is in contrast to other regions of the Netherlands where generally 30% of the population is from Non-Western descent (http://www.zorgatlas.nl). In our study sample only 34% were of Dutch origin and the majority of the non-western adolescents were from Turkish descent. However, we found no differences in intervention effects between adolescents from different ethnicities. Moreover, our study sample consists of obese adolescents seeking treatment. These adolescents generally had a higher level of psychopathology than those not seeking treatment [34]. Elevated levels of psychopathology are related with impaired HRQoL [35].

Another limitation is that our study had insufficient power to detect a significant difference in HrQol, since the power calculation was based on the primary outcome i.e. BMI-SDS. Therefore we focused on effect estimates and confidence intervals rather than statistical significance. Also a limitation of our trial is the low adherence to the Go4it program. Many adolescents were not motivated to attend the Go4it sessions every other week. Even after signing the informed consent form, some adolescents and their families were not willing to participate. We encouraged participant compliance by sending reminders using text messages and phone contact one week before sessions. The main reasons for not attending the Go4it sessions were the travel distance and the limited time of working parents and schoolchildren. Therefore, we recommend implementation of Go4it in a setting closer to the home environment, such as the child health care environment or school setting. The dropout rates of 20 and 44% at the 6 and 18-month follow-ups, respectively, are comparable to previous studies in obese adolescents (12-47%) [13,36-39].

In summary, obese adolescents experienced lower quality of life than their healthy peers.

Our low intensity, multidisciplinary, outpatient group treatment, Go4it, had small but beneficial effects on the health related quality of life of obese adolescents. This study shows that an intervention program targeting a healthy lifestyle among obese adolescents can improve their quality of life.

### Ethical approval

This study was approved by the Medical Ethical Committee of the VU University Medical Center Amsterdam. The adolescents as well as their parents gave written informed consent.

## Competing interests

The authors declare that they have no competing (financial) interests.

## Authors’ contributions

GH, PW, HD, MW, and MC provided support in the design of the study and contributed to the main ideas of this paper. GH conducted the statistical analyses and drafted the manuscript. PW, MC, MW and HD provided support during the development and implementation of the intervention. MC, MW and PW provided critical comments on the analyses and manuscript. All authors contributed to the interpretation of data and drafting of the manuscript. All authors read and approved the final manuscript.

## Pre-publication history

The pre-publication history for this paper can be accessed here:

http://www.biomedcentral.com/1471-2458/13/939/prepub
